# Extracorporeal Cardiopulmonary Resuscitation for Out‐of‐Hospital Cardiac Arrest in Adult Patients

**DOI:** 10.1161/JAHA.119.015291

**Published:** 2020-03-24

**Authors:** Akihiko Inoue, Toru Hifumi, Tetsuya Sakamoto, Yasuhiro Kuroda

**Affiliations:** ^1^ Department of Emergency, Disaster and Critical Care Medicine Faculty of Medicine Kagawa University Kagawa Japan; ^2^ Department of Emergency and Critical Care Medicine Hyogo Emergency Medical Center Kagawa Japan; ^3^ Department of Emergency and Critical Care Medicine St. Luke's International Hospital Tokyo Japan; ^4^ Department of Emergency Medicine Teikyo University Tokyo Japan

**Keywords:** extracorporeal cardiopulmonary resuscitation, out‐of‐hospital cardiac arrest, prevalence, pathophysiology, predictors, management, complications, Cardiopulmonary Arrest, Cardiopulmonary Resuscitation and Emergency Cardiac Care

## Abstract

Extracorporeal cardiopulmonary resuscitation (ECPR) followed by targeted temperature management has been demonstrated to significantly improve the outcomes of out‐of‐hospital cardiac arrest (OHCA) in adult patients. Although recent narrative and systematic reviews on extracorporeal life support in the emergency department are available in the literature, they are focused on the efficacy of ECPR, and no comprehensively summarized review on ECPR for OHCA in adult patients is available. In this review, we aimed to clarify the prevalence, pathophysiology, predictors, management, and details of the complications of ECPR for OHCA, all of which have not been reviewed in previous literature, with the aim of facilitating understanding among acute care physicians. The leading countries in the field of ECPR are those in East Asia followed by those in Europe and the United States. ECPR may reduce the risks of reperfusion injury and deterioration to secondary brain injury. Unlike conventional cardiopulmonary resuscitation, however, no clear prognostic markers have been identified for ECPR for OHCA. Bleeding was identified as the most common complication of ECPR in patients with OHCA. Future studies should combine ECPR with intra‐aortic balloon pump, extracorporeal membrane oxygenation flow, target blood pressure, and seizure management in ECPR.

**Nonstandard Abbreviations and Acronyms**

**AHA** American Heart Association

**BIS** bispectral index

**CPR** cardiopulmonary resuscitation

**CT** computed tomography

**ECMO** extracorporeal membrane oxygenation

**ECPR** extracorporeal cardiopulmonary resuscitation

**GWR** gray to white matter ratio

**IABP** intra‐aortic balloon pump

**ICH** intracerebral hemorrhage

**IL‐1** interleukin 1

**MRI** magnetic resonance imaging

**NIRS** near‐infrared spectroscopy

**NMDA** N‐methyl‐D‐aspartate

**NO** nitric oxide

**NOS** nitric oxide synthase

**NSE** neuron‐specific enolase

**OHCA** out‐of‐hospital cardiac arrest

**RBC** red blood cell

**ROS** reactive oxygen species

**ROSC** return of spontaneous circulation

**rSO**_**2**_ regional oxygen saturation

**SEP** somatosensory evoked potential

**TH** therapeutic hypothermia

**TNF‐α** tumor necrosis factor‐α

**TTM** targeted temperature management

**VA‐ECMO** venoarterial extracorporeal membrane oxygenation

**WBC** white blood cell

## Introduction

Extracorporeal cardiopulmonary resuscitation (ECPR) followed by targeted temperature management (TTM) has been demonstrated to significantly improve the outcomes of patients with out‐of‐hospital cardiac arrest (OHCA).^1^, ^2^, ^3^ Although the majority of the reports on ECPR management showed surprisingly good outcomes,^4^, ^5^ the principal components, such as the basic pathophysiology, and the critical adverse events, such as infection, hemorrhage, and ischemia, have not been examined.^6^, ^7^, ^8^ Recently, narrative and systematic reviews on extracorporeal life support in the emergency department have been finally published.^8^, ^9^ However, they were focused on the efficacy of ECPR, and no comprehensively summarized review on ECPR for OHCA in adult patients is available.

In this review, we aimed to clarify the prevalence, pathophysiology, predictors, management, and details of the complications of ECPR for OHCA, all of which have not been reviewed in previous literature, with the aim of facilitating understanding among acute care physicians.

## Definitions and Prevalence

### Definitions

ECPR can be defined as the implantation of venoarterial extracorporeal membrane oxygenation (VA‐ECMO) in patients who experience a sudden and unexpected pulseless condition secondary to cessation of cardiac mechanical activity.^10^ In Japan, the term percutaneous cardiopulmonary support has been commonly used, but it is similar to VA‐ECMO.

### Prevalence (Updated Worldwide Clinical Use of ECPR)

**Figure 1:**
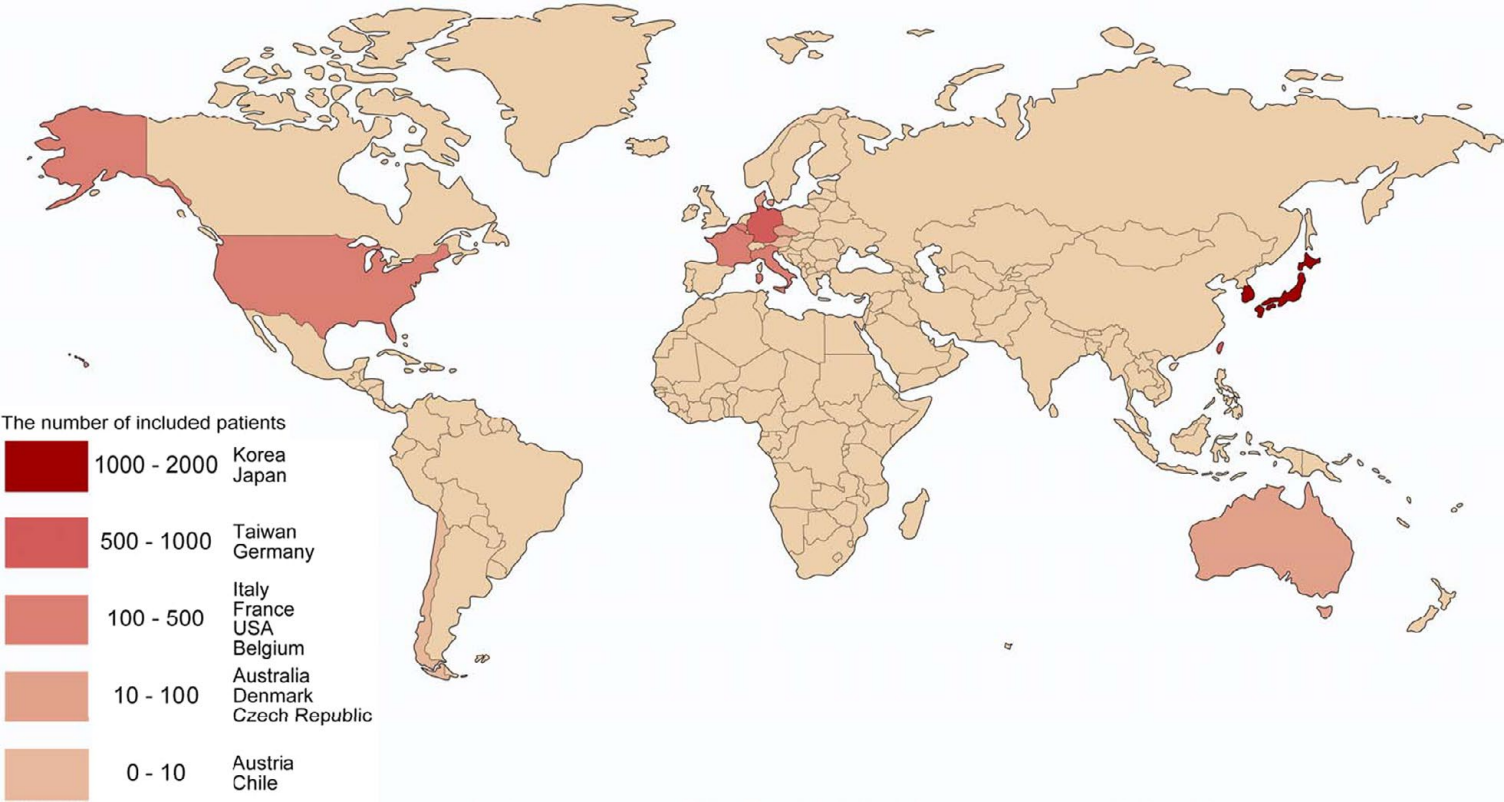
**Prevalence of extracorporeal cardiopulmonary resuscitation based on the published literature.**

We searched Medline via PubMed for full‐text clinical trials (to June 30, 2019) conducted on humans to retrieve the relevant articles for a literature review. The key search term that was used to identify the potential studies was “extracorporeal cardiopulmonary resuscitation.” Only literature written in English was included. The authors of the original reports were contacted if information was missing. If the information could not be obtained, we used the available data. The titles and abstracts of the retrieved records and full texts of potentially eligible records were screened independently by 2 reviewers. Any disagreements were resolved by discussion or consultation with a third author. Of the 269 citations identified with the search strategy, 61 studies on adult patients with OHCA treated with ECPR were finally examined (Figure 1).^4^, ^7^, ^11^, ^12^, ^13^, ^14^, ^15^, ^16^ In the current review, the studies examined by the Extracorporeal Life Support Organization Registry were excluded because of the unavailability of data for the individual countries, even after sending requests for retrieval to the authors.^6^, ^70^, ^71^ Notably, duplicate cases were not removed because of the unavailability of data sets. One of the limitations of this study was the possibility of duplicate cases because of the unavailability of some data sets. The leading countries in the field of ECPR were those in East Asia, such as Japan, Republic of Korea, and Taiwan, followed by European countries, such as Germany, France, and Italy, as well as the United States. In Japan, Sakamoto et al^4^ conducted a prospective observational study (ie, SAVE‐J [Study of Advanced Life Support for Ventricular Fibrillation with Extracorporeal Circulation in Japan]) to evaluate the effectiveness of ECPR and conventional cardiopulmonary resuscitation (CPR) for OHCA in adults with ventricular fibrillation/ventricular tachycardia on initial ECG, and concluded that a treatment bundle that included ECPR, therapeutic hypothermia, and intra‐aortic balloon pump (IABP) was associated with improved neurologic outcomes. Interestingly, a case report from Chile has recently been published.^15^ Therefore, the prevalence of ECPR has been rapidly spreading worldwide. At present, we are preparing for the next SAVE‐J II study to provide real‐world data about ECPR in Japan and to examine the indications, management, and prediction of neurologic outcomes (UMIN‐ID; UMIN000036490).

## Pathophysiology of Brain Injury and the Effects of ECPR After Cardiac Arrest

### Brain Injury After Cardiac Arrest

There are 2 mechanisms of hypoxic ischemic brain injury. The first is a primary brain injury that is caused by the cessation of oxygen delivery during cardiac arrest, and the other is secondary brain injury after reperfusion by resuscitation and/or return of spontaneous circulation (ROSC).^72^

When a patient goes into cardiac arrest, there is an immediate decrease in cerebral blood flow, which causes a reduction in cerebral oxygen delivery and leads to brain ischemia (primary brain injury). With the onset of cardiac arrest, the decreased cerebral oxygen delivery reduces the neuronal aerobic metabolism and cellular ATP production.^73^ This lack of energy leads to anaerobic metabolism, accumulation of lactic acid in the brain, and intracellular acidosis.^74^ With low oxygen delivery to the brain, the function of Na^+^ ion channels in the neurons is impaired, causing intracellular depolarization and cytotoxic edema. Moreover, intracellular calcium accumulation causes mitochondrial dysfunction and further reduction in ATP production.^75^ The sustained influx of intracellular Ca^2+^ ions through the N‐methyl‐D‐aspartate channel generates Ca^2+^‐dependent enzyme activation, failure of the cell membrane, production of reactive oxygen species, mitochondrial dysfunction, further reduction in ATP production, and finally cell death.^75^, ^76^

**Figure 2:**
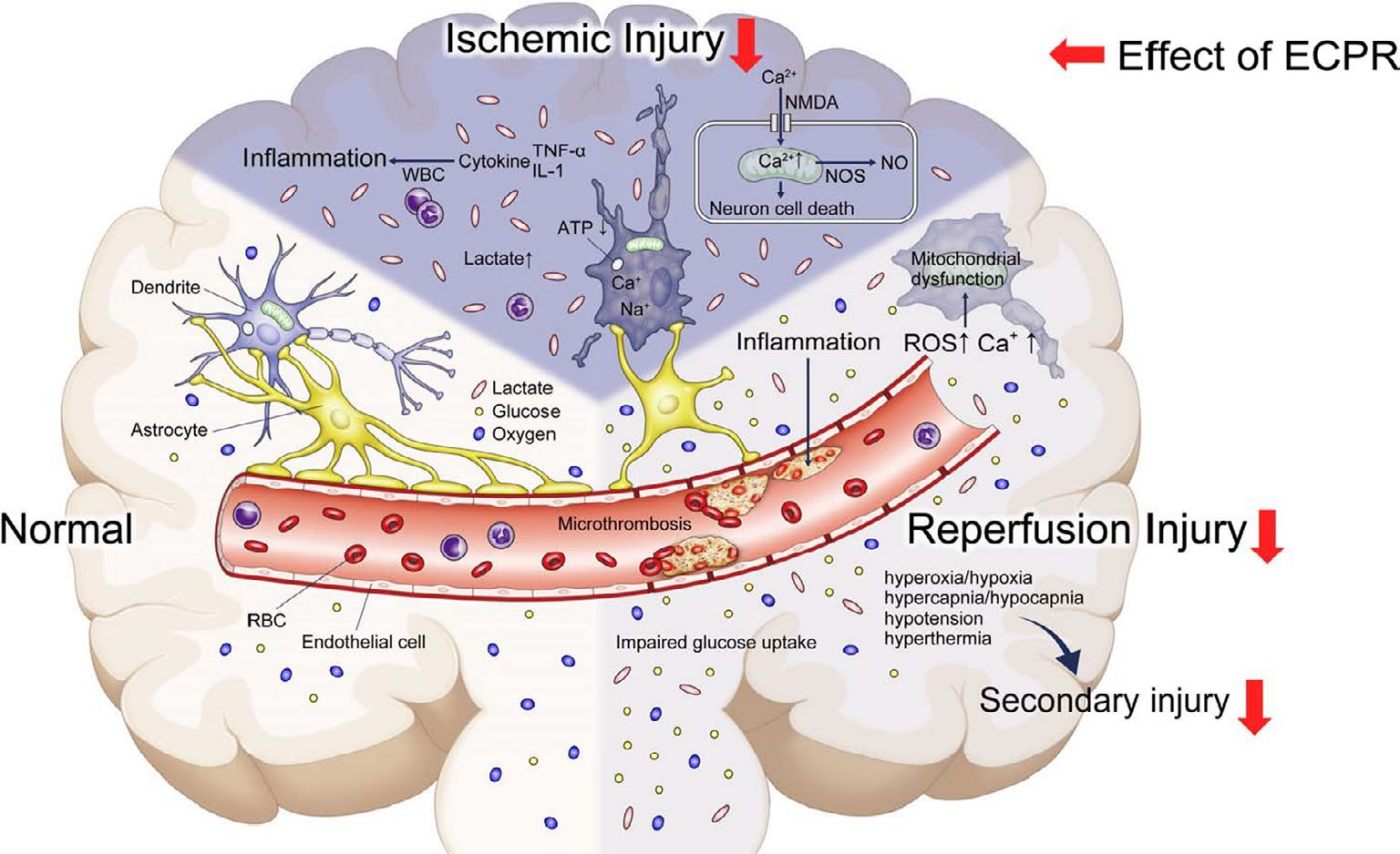
**Pathophysiology of brain injury and effects of extracorporeal cardiopulmonary resuscitation (ECPR) after cardiac arrest.** IL‐1 indicates interleukin 1; NMDA, N‐methyl‐D‐aspartate; NO, nitric oxide; NOS, nitric oxide synthase; RBC, red blood cell; ROS, reactive oxygen species; TNF‐α, tumor necrosis factor‐α; and WBC, white blood cell.

Secondary brain injury is caused by an imbalance between cerebral oxygen delivery and cerebral metabolic rate for oxygen after ROSC and may be caused by reperfusion, microcirculatory dysfunction, hyperoxia/hypoxia, hypercapnia/hypocapnia, hypotension, and hyperthermia, among other causes.^72^ Reperfusion injury, which can occur upon the restoration of blood flow to the ischemic organ, comprises free radical release, endothelial dysfunction, glutamate production, inflammatory cell activation, and intracellular Ca^2+^ accumulation.^77^ The significantly higher cerebral glucose concentration associated with unfavorable, rather than favorable, neurologic outcomes may be the consequence of impaired glucose uptake in the injured brain^78^ (Figure 2).

Postcardiac arrest syndrome encompasses brain injury, myocardial dysfunction, systemic ischemia/reperfusion response, and persistence of the precipitating pathology after cardiac arrest.^79^ The severity of these conditions after ROSC is based on the severity of the ischemic insult. Initiation of optimal CPR and rapid ROSC after the onset of cardiac arrest (ie, short duration of low blood flow) can reduce the severity of postcardiac arrest syndrome.

### Effects of ECPR for Patients With OHCA

VA‐ECMO pumps blood from the vein and returns it into an artery, thereby allowing the circulation of oxygenated and decarboxylated blood in the body even in the presence of severe heart failure or cardiac arrest. During ECPR, initiation of extracorporeal flow can provide systemic organ perfusion, despite the absence of ROSC. ECPR has the potential to alter some of the factors after cardiac arrest. Compared with conventional CPR, ECPR has been shown to increase coronary perfusion pressure,^80^ the rate of ROSC,^80^ and the rate of successful defibrillation.^81^ Early reperfusion has been shown to improve hemodynamic status, such as carotid blood flow and the rate of ROSC, along with a decrease in the size of the myocardial infarct.^82^ In general, extracorporeal membrane oxygenation (ECMO) has been associated with an increase in inflammatory mediators, such as endotoxins, oxygen‐derived free radicals, and cytokines.^83^ During cardiac arrest, a low‐flow state is not necessarily associated with a decrease in the inflammatory cascade.^84^

Conventional CPR can provide only 25% to 30% of the cardiac output,^85^ whereas ECPR can provide sufficient perfusion to organs, including the brain,^86^ and can reduce the duration of the low‐flow status. Therefore, compared with conventional CPR, ECPR can reduce the risk of developing primary brain injury (Figure 2, red arrow). From the early phase after initiation, ECPR can provide stable oxygenation, controlled carbon dioxide levels, and sufficient systemic organ perfusion. Moreover, the use of a heat exchanger can rapidly achieve and sustain TTM, including therapeutic hypothermia, via the VA‐ECMO circuit.^87^ Therefore, ECPR may reduce the chances of reperfusion injury and deterioration to a secondary brain injury (Figure 2, red arrow).

Another advantage of ECPR is the facilitation of coronary interventions, even in patients with sustained ventricular fibrillation,^88^ because VA‐ECMO provides stable systemic perfusion. Therefore, ECPR has been considered to provide a bridge to the subsequent diagnosis and treatment of the underlying cause of cardiac arrest and, possibly, to contribute to increased survival rates and good neurologic outcomes. However, there is a need for further examination of detailed data on the association of ECPR with postcardiac arrest syndrome, including the reperfusion and systemic inflammatory responses, along with the various underlying microvascular and cellular pathophysiologic processes.

## Prediction of Outcomes

The guidelines of the American Heart Association (AHA) recommend the use of physical examination, electrophysiologic modalities, imaging modalities, and blood markers for the prediction of neurologic outcomes after cardiac arrest.^89^ Furthermore, they recommend that the earliest time to prognosticate a poor neurologic outcome using clinical examination is 72 hours after cardiac arrest in patients not treated with TTM and 72 hours after return to normothermia in patients treated with TTM.^89^ In the setting of ECPR for OHCA in adult patients, several studies examining the predictors of neurologic outcomes were reported.

### Physical Examination

Physical examination may be helpful for predicting the neurologic outcomes after cardiac arrest. In a prospective observational cohort of patients with ECPR (n=53), Maekawa et al^1^ analyzed and reported the association of pupil diameter upon hospital arrival with neurologic outcomes (adjusted hazard ratio, 1.39 per 1 mm increase; 95% CI, 1.09–1.78 [*P*=0.008]). Receiver operating characteristic analysis showed a pupil diameter of <6 mm as the optimal cutoff point for predicting favorable outcomes, with an area under the curve value of 0.87 (95% CI, 0.75–0.98); sensitivity and specificity of 100% and 59%, respectively; and positive and negative predictive values of 31% and 100%, respectively.^1^

### Computed Tomography Imaging

With regard to imaging modalities, Lee et al^56^ reported the utility of the gray to white matter ratio (GWR) on brain computed tomography within 1 hour on pump during ECPR for patients with OHCA (n=30). The cutoff values with 100% specificity for the prediction of poor outcomes were 1.23 for GWR‐average (sensitivity: 76%), 1.24 for GWR‐basal ganglia (sensitivity: 88.0%), 1.22 for GWR‐cortical (sensitivity: 64%), and 1.21 for GWR‐simplified (sensitivity: 76%).

### Bispectral Index and Near‐Infrared Spectroscopy

A report by Jouffroy et al^90^ of patients with ECPR (n=46) showed that a bispectral index of <30 under mild therapeutic hypothermia had a sensitivity of 96%, specificity of 82%, positive predictive value of 90%, and negative predictive value of 93% for the prediction of brain death during intensive care unit hospitalization. The area under the curve for the receiver operating characteristic analysis of the initial bispectral index value under mild therapeutic hypothermia was 0.86 (0.73–0.98). In a retrospective study with a small sample (n=16), Ehara et al^41^ reported on cerebral regional oxygen saturation by near‐infrared spectroscopy. Specifically, they showed that cerebral regional oxygen saturation significantly increased during ECPR in the poor neurologic outcome group (*P*<0.01) but not in the good neurologic outcome group (*P*=0.88); therefore, the absence of a significant improvement in cerebral regional oxygen saturation during ECPR might have led to favorable neurologic outcomes.^41^

### Time

In patients with cardiac arrest, time to treatment has been recognized as the main predictor of survival.^91^ Although the provided time to treatment is longer with ECPR than with conventional CPR,^27^ the survival rate declines with further delay in treatment.^27^ In a patient with ECPR, time to initiation of VA‐ECMO could be a predictor of survival and good neurologic outcome.^37^, ^40^, ^92^ Otani et al^37^ retrospectively analyzed witnessed patients with OHCA who received ECPR (n=135). In multiple logistic analyses, shorter low‐flow time was significantly associated with favorable neurologic outcomes (odds ratio, 0.88; 95% CI, 0.82–0.94). The area under the receiver operating characteristic curve for low‐flow time was 0.80 (95% CI, 0.70–0.89), and a cutoff value of 58 minutes corresponded to a sensitivity of 0.25 and a specificity of 1.0. A systematic review reported that a longer low‐flow time was associated with poor outcome (geometric mean ratio, 0.90; 95% CI, 0.81–0.99).^93^ Previous reports have described the effect of prehospital ECPR on the minimization of the low‐flow time.^20^, ^94^ Moreover, a randomized trial comparing prehospital ECMO with in‐hospital ECMO for patients with OHCA is currently ongoing. The APACAR2 trial (A Comparative Study Between a Pre‐hospital and an In‐hospital Circulatory Support Strategy [ECMO] in Refractory Cardiac Arrest) (NCT02527031, ClinicalTrials.gov) in France is randomizing patients with refractory cardiac arrest to receive an insertion of ECMO either in a prehospital setting or in an in‐hospital setting, with an estimated enrollment of 210 patients.

### Laboratory Examination

In a systematic review and meta‐analysis by Debaty et al,^93^ the authors reported that higher arterial pH and lower serum lactate concentration upon hospital admission were associated with better outcomes of ECPR after OHCA. The mean difference in arterial pH upon admission was 0.12 (95% CI, 0.03–0.22; *P*=0.01) for 79 patients with favorable outcomes (7.12; 95% CI, 7.03–7.21), relative to 313 patients with unfavorable outcomes (6.99; 95% CI, 6.95–7.04). The mean difference in serum lactate concentration upon admission was −3.52 mmol/L (95% CI, −5.05 to −1.99; *P*<0.001) for 66 patients with favorable outcomes (9.97 mmol/L; 95% CI, 8.13–11.81), relative to 288 patients with unfavorable outcomes (13.26 mmol/L; 95% CI, 10.85–15.67).^93^

To date, many studies have examined the predictors of neurologic outcomes in patients with OHCA who received conventional CPR. These reported predictors include the absence of pupillary light and corneal reflexes, persistent absence of reactivity and persistent burst suppression on electroencephalogram, absence of N20 somatosensory evoked potential wave, GWR on computed tomography, diffusion on brain magnetic resonance imaging, neuron‐specific enolase, and S‐100B (Table 1).^89^, ^95^, ^96^

**Table 1:** Prognostication of Patients With Postcardiac Arrest After ECPR and Conventional CPR

	ECPR for OHCA	Conventional CPR for OHCA^95^
Physical examination	Pupil diameter ≥6 mm upon hospital arrival^1^	Absence of pupillary light reflex, corneal reflexes, and motor responses to pain Status myoclonus
Imaging	GWR on CT at ≤1 h after pump^56^	GWR on CT MRI (apparent diffusion coefficient)
Electrophysiology	BIS value <30 under TH^90^ rSO_2_ by NIRS^41^	SEP (bilateral absence of N20) Electroencephalography (absence of electroencephalography reactivity, status epilepticus, and burst suppression)
Laboratory examination	Arterial pH value^93^ Serum lactate levels^93^	NSE
Others	Time to initiation of ECPR >58 min^37^	

Patients in deep coma and those with confounders, such as hypotension, hypothermia, hypoxia, and presence of residual drugs for sedation, analgesia, and neuromuscular blockade, were excluded. BIS indicates bispectral index; CPR, cardiopulmonary resuscitation; CT, computed tomography; ECPR, extracorporeal cardiopulmonary resuscitation; GWR, gray to white matter ratio; MRI, magnetic resonance imaging; NIRS, near‐infrared spectroscopy; NSE, neuron‐specific enolase; OHCA, out‐of‐hospital cardiac arrest; rSO_2_, regional oxygen saturation; SEP, somatosensory evoked potential; and TH, therapeutic hypothermia.

## Management

### Targeted Temperature Management

The AHA guidelines recommend TTM (32–36°C) after ROSC in patients with cardiac arrest (grade I).^97^ In the setting of ECPR for patients with OHCA, TTM was reported to be associated with good neurologic outcomes.^37^, ^61^, ^87^ In an observational study (n=135), Otani et al^37^ reported that 34°C TTM was significantly associated with favorable outcomes in the multivariate analysis (odds ratio, 4.51; 95% CI, 1.19–17.06). In a prospective observational study (n=171), Nagao et al^87^ reported that early induction of hypothermia (collapse to 34°C interval) was an independent predictor of favorable outcome, with an adjusted odds ratio of 0.99 (95% CI, 0.98–1.00; *P*=0.035).

In a pig model, mild therapeutic hypothermia (33°C) was superior to controlled normothermia (36.8°C) in the maintenance of blood pressure, cerebral oxygenation, organ protection, and oxidative stress suppression following cardiac arrest.^98^ On the other hand, normothermia (38.0°C) and hypothermia (32.0–33.0°C) were equal in resuscitation success rates, postarrest cardiac function, and magnitude of myocardial injury.^99^

To date, there have been no randomized controlled trials on TTM in patients who have undergone ECPR. Therefore, the optimal temperature (hypothermia versus normothermia), duration, and complications related to TTM have not been discussed.

### Intra‐Aortic Balloon Pump

During peripheral VA‐ECMO, the arterial perfusion is retrograde and the increasing afterload on the left ventricle may lead to pulmonary edema. Diastolic augmentation during IABP has been shown to increase the mean arterial pressure^100^ and to significantly increase the antegrade mean flow in the middle cerebral artery.^101^ IABP had been considered to provide left ventricular unloading in VA‐ECMO. Using propensity score matching of cases from a national inpatient database, Aso et al^102^ reported that IABP with peripheral VA‐ECMO upon admission was associated with improved mortality and successful weaning from VA‐ECMO in patients with cardiac shock. These studies mainly included patients in cardiac shock, not those with cardiac arrest, which may represent a distinct patient group. Moreover, the timing of the insertion of IABP with VA‐ECMO was not discussed.

In a pig model of ventricular fibrillation in subjects that underwent femorofemoral cannulation, the addition of IABP to VA‐ECMO^86^ did not significantly change the carotid flow velocity from baseline (femorofemoral ECMO 90.3% versus femorofemoral ECMO+IABP 81.8%, *P*= 0.16), but it significantly decreased the coronary flow velocity from baseline (femorofemoral ECMO 90.0% versus femorofemoral ECMO+IABP 60.7%, *P*=0.004). Moreover, there were no significant changes in the oxygen saturation from baseline in the brain (femorofemoral ECMO 93.8% versus femorofemoral ECMO+IABP 91.9%, *P*=0.74) and in the peripheral organs (femorofemoral ECMO 92.1% versus femorofemoral ECMO+IABP 94.5%, *P*=0.46).^86^

To date, most of the ECPR studies included a combination of VA‐ECMO with IABP.^3^ However, the association between IABP and neurologic outcomes, as well as the timing of IABP insertion, was not examined. The neuroprotective benefits of IABP remain unknown. Moreover, several studies have reported the use of other mechanical techniques, such as Impella (ABIOMED) or TandemHeart (CardiacAssist, Inc), and unloading techniques, such as left ventricular vent, atrial septostomy, and pigtail in the left ventricle, to reduce ECMO‐associated pulmonary edema during cardiogenic shock.^103^, ^104^ Despite the absence of prospective randomized data, a previous study reported an association between left ventricular unloading and decreased mortality in patients treated with VA‐ECMO for cardiogenic shock.^105^ However, there are no studies regarding the concomitant application of these techniques with ECPR in patients with OHCA.

### Hemodynamic Strategy

In a pig model, a low‐blood‐flow strategy (30–35 mL/kg per minute versus 65–70 mL/kg per minute) during the first 6 hours of resuscitation was reported to be associated with lower lactate clearance (*P*=0.04) and lower cerebral blood flow (*P*<0.005), but it had no benefits.^84^ In another study using a pig model, high and low blood pressure targets in the first hours of ECPR had no significant differences in terms of any hemodynamic improvement and in the amount of infused fluid.^106^ There had been no human data on the hemodynamic strategy for ECPR.

In the field of intensive care, there are limited data on the other management strategies after ROSC in patients with OHCA who have received ECPR. In particular, combination with IABP, ECMO flow, target blood pressure, and seizure management need to be studied in the future.

## Complications

**Figure 3:**
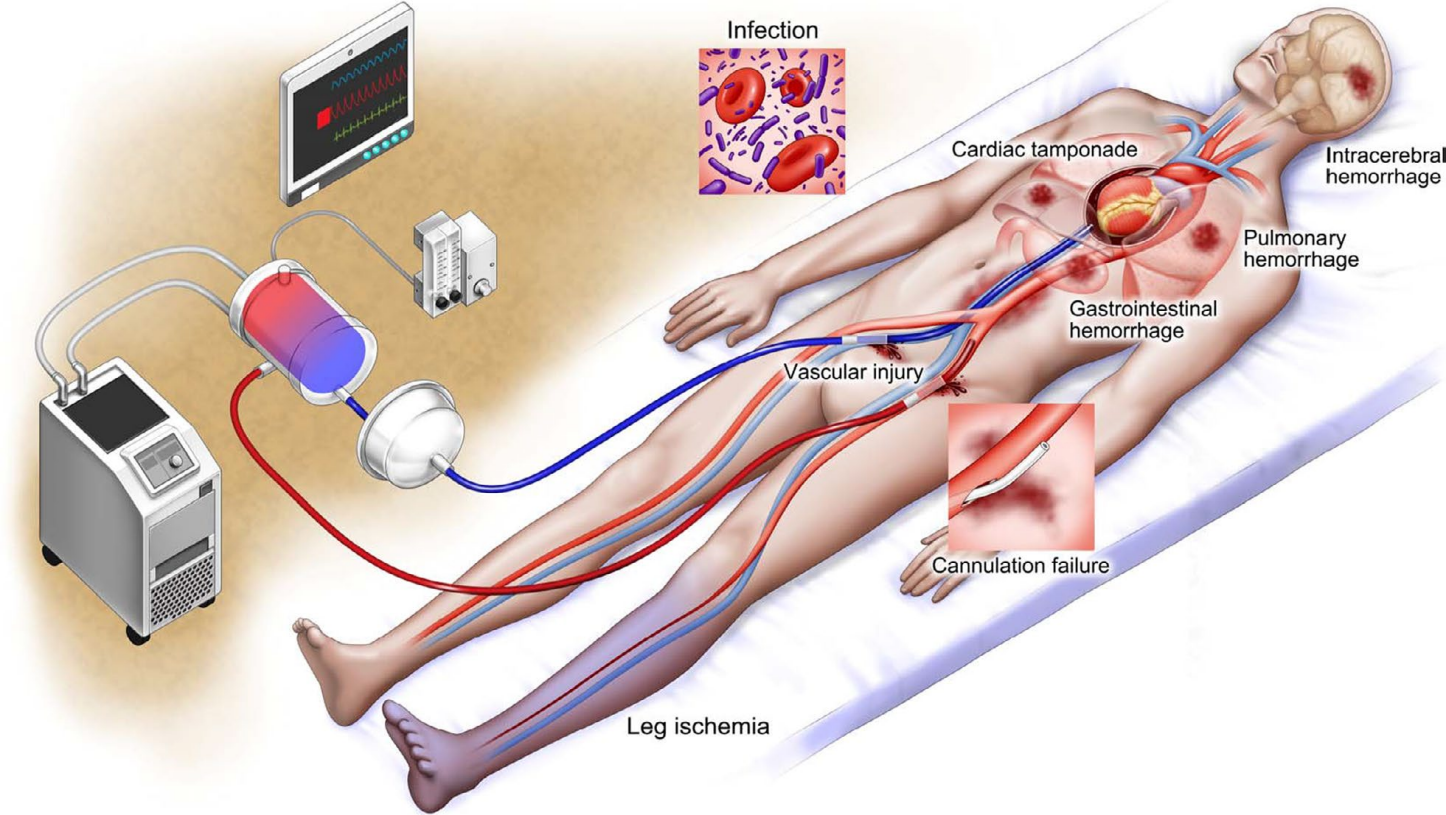
**Complications related to extracorporeal cardiopulmonary resuscitation for patients with out‐of‐hospital cardiac arrest.**

ECPR‐related complications are important because of the high risk of death and the associated poor outcome.^61^ We summarized the reported complications related to ECPR for patients with OHCA (Table 2, Figure 3).^1^, ^7^, ^55^, ^61^, ^92^, ^107^, ^108^, ^109^

**Table 2:** Details of the Complications Related to ECPR for Patients With OHCA

Authors	Design	Complications	Cannulation Strategy
Leick et al (2013)^92^	Retrospective (n=28)	Bleeding 32% (all at the cannulation site), leg ischemia 4%	All patients were directly transferred to the catheterization laboratory
Maekawa et al (2013)^1^	Prospective observational cohort (n=53)	Bleeding 32.7%, leg ischemia 15.4%, unsuccessful cannulation 1.9%, infection 7.7%, compartment syndrome 1.9%	
Kim et al (2014)^61^	Retrospective (n=55)	Significant bleeding 27.3%, leg ischemia 6.8%, circuit failure 0%, ICH/stroke 2.3%	
Champigneulle et al (2015)^107^	Retrospective (n=43)	Unsuccessful cannulation 51.2%	
Lee JJ et al (2016)^108^	Retrospective (n=23)	Bleeding 13%, leg ischemia 8.7%, unsuccessful cannulation 0%, circuit failure 0%, ICH/stroke 17.4%, sepsis 21.7%	
Pozzi et al (2016)^109^	Retrospective (n=68)	Cannulation failure 6%	
Ha et al (2017)^55^	Retrospective (n=35)	Bleeding 37%, leg ischemia 3%	Fluoroscopic guidance 40%
Kashiura et al (2017)^110^	Retrospective (n=73)	Bleeding 8.2%, vascular injury 4.1%, change to surgical approach 5.6%, aberrant placement of cannula 4.1%, hematoma 21%	Ultrasound alone, 68%; both fluoroscopy and ultrasound, 32%
Ohtani et al (2018)^7^	Retrospective (n=102)	Bleeding 70% (cannulation site 49%, thorax 28%, gastrointestinal tract 24%, abdomen 14%, alveolar hemorrhage 10%, nasal bleeding 7%)	

ECPR indicates extracorporeal cardiopulmonary resuscitation; ICH, intracerebral hemorrhage; and OHCA, out‐of‐hospital cardiac arrest.

### Bleeding

Bleeding is the most common complication of ECPR in patients with OHCA (8%–70%).^1^, ^7^, ^55^, ^61^, ^92^, ^108^, ^110^ Anticoagulation for the prevention of thrombosis in VA‐ECMO can predispose to easy bleeding; this can make the procedure relatively difficult, considering the fact that emergent cannulation is performed under resuscitation. Bleeding can occur in the cannulation site, such as in cases of vessel injury and retroperitoneal hemorrhage, and in systemic organs, such as in cases of intracerebral, gastrointestinal, and pulmonary hemorrhage.^9^ CPR‐related complications can result in bleeding, such as hemothorax, abdominal hemorrhage, and cardiac tamponade.^111^ Otani et al^7^ reported that, among 133 patients with OHCA who received ECPR, the most frequent bleeding site was the cannulation site (49%). In that study, bleeding occurred in the thorax and abdomen of 28% and 14%, respectively, of the patients who had CPR‐related complications. To prevent complications, such as bleeding or vessel injury, avoidance of excess vessel dilatation and manipulation against resistance during cannulation has been recommended.^9^

### Unsuccessful Cannulation

The other common complication related to ECPR is unsuccessful cannulation (2%–51%).^1^, ^107^, ^108^, ^109^, ^110^ Compared with cannulation under guidance by ultrasound alone, the combination of ultrasound‐ and fluoroscopy‐guided cannulation was reported to have a lower incidence of cannulation‐related complications, including bleeding and inadequate placement of the cannula (36% versus 8.7%, *P*=0.022).^110^ However, in some situations, emergent cannulation during CPR is performed in the emergency department without fluoroscopic imaging.

### Limb Ischemia

Limb ischemia after femoral cannulation for ECMO may occur because of the obstruction of flow by the arterial cannula and may necessitate leg amputation. Studies on ECPR for OHCA have described limb ischemia rates between 3% and 15%.^1^, ^55^, ^61^, ^92^, ^108^ To prevent this complication, a limb reperfusion cannula is required. If distal arterial flow to the leg is inadequate, distal retrograde perfusion of the superficial femoral artery or the posterior tibial artery would be needed.^112^

### Infection

Patients who have undergone ECPR were reported to have an 8% to 22% risk of developing cannulation site infection or cannula‐related infection.^1^, ^108^

Because ECPR requires emergent cannulation during an ongoing CPR, it is more difficult and has a relatively high risk for complications. Simulation training can enable emergency medicine providers to rapidly and safely initiate ECPR.^113^

## ECPR Versus Conventional CPR

Although there are no published randomized trials comparing ECPR with conventional CPR, several observational studies have reported that compared with conventional CPR, ECPR improved the survival rates and neurological outcomes of patients with OHCA.^1^, ^4^, ^61^, ^114^ Sakamoto et al^4^ conducted a prospective observational study (ie, the SAVE‐J study) involving an intention‐to‐treat analysis. They reported that the proportions of patients with a cerebral performance category of 1 or 2 in the ECPR and non‐ECPR groups were 12.3% and 1.5%, respectively, at 1 month (*P*<0.0001) and 11.2% and 2.6%, respectively, at 6 months (*P*=0.001). Using propensity score matching of cases from a prospective cohort, Kim et al^61^ reported that the rate of a favorable neurological outcome at 3 months postcardiac arrest was significantly higher in the matched ECPR group that had received CPR for a duration of ≥21 minutes than in the matched conventional CPR group (15.4% versus 1.9%, *P*=0.031). Moreover, some recent systematic reviews have demonstrated trends that link ECPR with improved survival and good neurologic outcomes.^2^, ^3^, ^8^ However, the effects of ECPR have not been clearly elucidated, indicating the need for larger and more randomized trials.

Several randomized trials comparing ECPR with conventional CPR for patients with OHCA are currently ongoing. The INCEPTION (Early Initiation of Extracorporeal Life Support in Refractory OHCA) trial (NCT03101787 ClinicalTrials.gov) in the Netherlands is randomizing patients with OHCA to either an initiation of ECPR or conventional CPR. The primary outcome for the study is 30‐day survival rate with favorable neurological status defined as cerebral performance category scale 1 to 3, and estimated enrollment of 110 patients. The ECPB4OHCA (Emergency Cardiopulmonary Bypass for Cardiac Arrest) trial (NCT01605409 ClinicalTrials.gov) in Austria is also comparing ECPR with conventional CPR, and the primary outcome is the rate of ROSC during 48 hours with a sample size of 40 patients. The EROCA Study (NCT03065647 ClinicalTrials.gov) in the United States is currently randomizing patients with OHCA to the expedited transport to an emergency department capable of initiating ECPR or standard care. The primary outcome is emergency department arrival interval, ECPR initiation interval, and a planned sample size of 30 patients. The purpose of this study is to examine the feasibility and potential benefit of early transport to an ECPR‐capable emergency department.

## Conclusions

In this review, we have provided comprehensive information about ECPR in adult patients with OHCA. The leading countries in the field of ECPR are those in East Asia followed by those in Europe and the United States. ECPR may reduce the risks of reperfusion injury and deterioration to secondary brain injury. Unlike conventional CPR, however, no clear prognostic markers have been identified for ECPR for OHCA. Bleeding was identified as the most common complication of ECPR in patients with OHCA. Future studies should combine ECPR with IABP, ECMO flow, target blood pressure, and seizure management in ECPR. Further detailed examination with a large number of patients (eg, SAVE‐J II study) is needed to draw robust conclusions about ECPR.

## Sources of Funding

This study was supported by the Japan Society for the Promotion of Science (JSPS) KAKENHI (Grant‐in‐Aid for Scientific Research [C]) grant number JP19K09419.

## Disclosures

None.
